# Searching for a Home Port in a Polyvectic World: Molecular Analysis and Global Biogeography of the Marine Worm *Polydora hoplura* (Annelida: Spionidae)

**DOI:** 10.3390/biology12060780

**Published:** 2023-05-27

**Authors:** Vasily I. Radashevsky, Vasily V. Malyar, Victoria V. Pankova, Jin-Woo Choi, Seungshic Yum, James T. Carlton

**Affiliations:** 1 National Scientific Center of Marine Biology, Far Eastern Branch of the Russian Academy of Sciences, 17 Palchevsky Street, Vladivostok 690041, Russia; 2 Blue Carbon Research Center, Seoul National University, Seoul 08826, Republic of Korea; 3 Ecological Risk Research Division, Korea Institute of Ocean Science & Technology, Geoje 53201, Republic of Korea; 4 Coastal and Ocean Studies Program, Williams College-Mystic Seaport, Mystic, CT 06355, USA

**Keywords:** polychaete, biological invasions, distribution, aquaculture, vessel biofouling, ballast, molecular systematics

## Abstract

**Simple Summary:**

Transoceanic shipping and global development of aquaculture are the main vectors for the introduction of marine organisms, as adults or their larvae, to new remote locations. Recent invasions by large species may be well known and documented, while older and smaller-bodied invasions are often hidden and more difficult to detect. In the present study, we investigated, using molecular methods, marine worms that bore into the shells of commercial molluscs on four continents. We have identified them as *Polydora hoplura*, a species originally described from Italy. The highest genetic diversity was detected in South African population. While high genetic diversity is often regarded as indicative of a species’ natural range, our analysis of the worldwide discovery of *P. hoplura* calls into question its natural distribution in South Africa. The high genetic diversity of *P. hoplura* in this region may be the result of a complex dispersal history by ships and aquaculture. We tentatively propose the Northwest Pacific, or at the most the Indo–West Pacific, as its home region, not the Atlantic Ocean or the Eastern Pacific Ocean, and call for further exploration of this hypothesis.

**Abstract:**

The spionid polychaete *Polydora hoplura* Claparède, 1868 is a shell borer widely occurring across the world and considered introduced in many areas. It was originally described in the Gulf of Naples, Italy. Adult diagnostic features are the palps with black bands, prostomium weakly incised anteriorly, caruncle extending to the end of chaetiger 3, short occipital antenna, and heavy sickle-shaped spines in the posterior notopodia. The Bayesian inference analysis of sequence data of four gene fragments (2369 bp in total) of the mitochondrial *16S* rDNA, nuclear *18S*, *28S* rDNA and *Histone 3* has shown that worms with these morphological features from the Mediterranean, northern Europe, Brazil, South Africa, Australia, Republic of Korea, Japan and California are genetically identical, form a well-supported clade, and can be considered conspecific. The genetic analysis of a *16S* dataset detected 15 haplotypes of this species, 10 of which occur only in South Africa. Despite the high genetic diversity of *P. hoplura* in South Africa, we tentatively propose the Northwest Pacific, or at the most the Indo–West Pacific, as its home region, not the Atlantic Ocean or the Eastern Pacific Ocean. The history of the discovery of *P. hoplura* around the world appears to be intimately linked to global shipping commencing in the mid-19th century, followed by the advent of the global movement of commercial shellfish (especially the Pacific oyster *Magallana gigas*) in the 20th century, interlaced with continued, complex dispersal by vessels and aquaculture. Given that *P. hoplura* has been detected in only a few of the 17 countries where Pacific oysters have been established, we predict that it may already be present in many more regions. As global connectivity through world trade continues to increase, it is likely that novel populations of *P. hoplura* will continue to emerge.

## 1. Introduction

Polydoriases, “diseases” caused by shell-boring marine worms of the genus *Polydora* Bosc, 1802 and related genera (members of the tribe Polydorini Benham, 1896, collectively called polydorins), are often a plague in the cultivation of marine molluscs, especially oysters, abalone and clams [1,2,3,4,5]. Despite a long history of studies of these worms, the specific identification of the worms involved often remains problematic. Transported and introduced globally with aquaculture products [6,7,8] and other vectors, polydorins were often poorly described, misidentified or mistakenly redescribed as new species. Genetic characteristics are very helpful and sometimes crucial for the identification of shell-boring polydorins when morphological features are few and ambiguous [9]. In particular, specimens from the type locality of a species must be morphologically and genetically characterized in order to reliably anchor the name of a species to a precise lineage. While the type locality of a species is not necessarily within its native range [8,10], it often remains a fundamental aspect of understanding a species concept.

For many years most of the shell borers (also known as mud worms) around the globe were referred to as *Leucodore ciliatus* Johnston, 1838 (=*Polydora ciliata*), originally described from Berwick Bay (North Sea, Scotland). Radashevsky et al. [11], however, suggested that the systematic status and the mode of life of this species had been misinterpreted and that the global reports most likely include a complex of species.

**Figure 1:**
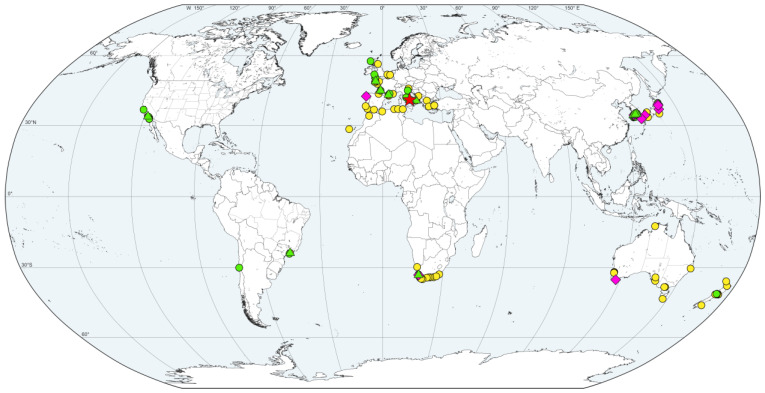
Map showing worldwide records of *Polydora hoplura* (see Supplementary Materials Tables S1 and S3). *Polydora hoplura*: red star—type locality: Gulf of Naples, Italy; green circles—worms identified based on the morphology in the present study; yellow circles—worms identified based on the morphology by other authors and not verified in the present study; green triangles—specimens sequenced in the present study; magenta rhombi—specimens sequenced by other authors.

As part of the global puzzle of mud worm identification, the spionid polychaete *Polydora hoplura* Claparède, 1868 is a shell borer widely occurring across the world and considered introduced in many areas. It was originally described from the Gulf of Naples (Tyrrhenian Sea, Italy) and later reported from the Mediterranean, Atlantic Europe, South Africa, New Zealand, Australia, Brazil and California (Figure 1; see reviews by Radashevsky et al. [12] and Radashevsky and Migotto [13]). Earlier records of *P. hoplura* from Kuwait [14] represent an undescribed species (Radashevsky unpublished). Identical-looking worms from Yamada Bay (Honshu, Japan) were described as a new species, *Polydora uncinata* Sato-Okoshi, 1998, and under this name reported from enclosed aquaculture operations in Chile [15], Republic of Korea [16] and Western Australia [17]. *Polydora hoplura* from Italy and *Polydora uncinata* from Japan were compared and found to be identical, and thus, *P. uncinata* was treated as a junior synonym of *P. hoplura* by Radashevsky et al. [12]. *Polydora hoplura*/*uncinata* from Japan, Australia, South Africa and Republic of Korea were sequenced by Sato-Okoshi and Abe [17], Teramoto et al. [18], David et al. [19], Williams et al. [9,20], Sato-Okoshi et al. [21], Lee et al. [22] and Abe and Sato-Okoshi [23]. Single *16S* sequence of an individual from Galicia (NW Spain) was provided by Almón et al. [24]. Genetic data for *P. hoplura* from the Mediterranean have remained unavailable.

We collected new and re-examined museum specimens of *Polydora* worms with morphological characteristics of *P. hoplura* worldwide, including its Gulf of Naples type locality. The purpose of the present study was to provide sequence data for worms from the type and other localities and verify by molecular analysis the conspecificity of disjunct populations. Based on the results of the genetic analysis, we further attempt to dissect and elucidate aspects of the global history of *P. hoplura* and ask whether the type locality is within the native range of this species.

## 2. Materials and Methods

### 2.1. Material

Collections were made in Italy (Tyrrhenian and Ionian Seas), France (both Atlantic and Mediterranean coasts), Republic of Korea (East Sea), USA (California), Brazil (São Paulo) and Chile (Figure 1). We collected bivalves, gastropods, barnacles, sponges and coralline algae from the intertidal zone manually and from shallow water using SCUBA equipment, grabs and trawls. Live *Polydora* worms were removed from infested shells or other substrata with a hammer and pliers, relaxed in isotonic magnesium chloride and then examined and photographed using light microscopes equipped with digital cameras. For molecular analysis, worm fragments were preserved in 95% ethanol.

After morphological examination in life, worms were fixed in 10% formalin solution, rinsed in fresh water, and transferred to 70% ethanol. Formalin-fixed specimens stored in ethanol were stained with Methylene Green (MG) to reveal specific staining pattern. We mainly followed the procedure described by Winsnes [25] (p. 19). The staining solution was made by adding MG to 70% ethanol to give a dark green color; thus, specimens in a dish could not be seen when covered by the solution. Specimens were soaked for 2–3 min and then moved to another dish containing clean 70% ethanol, where they were left to destain for 2–3 min until the excess color had disappeared. Stained specimens were examined and photographed using light microscopes equipped with digital cameras. Images of multiple focal layers were stacked using Zerene Stacker 1.04 software. Images of parts of worms were stitched into panoramas using PTGui 12.8 software. After complete examination, specimens were deposited in the polychaete collection of the Museum of the A.V. Zhirmunsky National Scientific Center of Marine Biology (MIMB), Vladivostok, Russia.

We also examined museum samples of various *Polydora* species worldwide, and collected for molecular comparison *Polydora brevipalpa* Zachs, 1933 from Peter the Great Bay, the Sea of Japan, Russia. Additional formalin- and/or ethanol-fixed *Polydora* specimens from Italy (Tyrrhenian Sea), France (Arcachon Bay and Gulf of Lion), Croatia (Adriatic Sea), Brazil (São Paulo), New Zealand, South Africa and California were provided by Maria Cristina Gambi, Nicolas Lavesque, Céline Labrune, Barbara Mikac, João Nogueira, Sean Handley, Carol Simon and Sergey Nuzhdin.

To map the distribution of *P. hoplura*, we considered reliable records made by earlier authors based on morphological features and records by Sato-Okoshi and Abe [17], Teramoto et al. [18], David et al. [19], Williams et al. [9,20], Sato-Okoshi et al. [21], Lee et al. [22], Abe and Sato-Okoshi [23] and Almón et al. [24] based on genetic data. Complete information on newly collected material, museum samples examined during this study and by other authors and records by other authors for which no museum deposits were noted are provided in Supplementary Materials Tables S1 and S3. A list of the museums and other collections (and their acronyms) holding the examined or reported specimens of *P. hoplura* is provided in Supplementary Materials Table S2. The complete bibliography of the records provided by other authors and noted in Supplementary Materials Tables S1, S3 and S5 is provided in Supplementary Materials Table S6.

When no coordinates were provided for sampling sites from other studies, they were estimated using Google Earth Pro 7.3.6.9345 according to the original descriptions of the locations. Sampling locations of *P. hoplura* noted in Supplementary Materials Tables S1 and S3 are plotted on maps using QGIS 3.20.0 software and the geodata provided by the OpenStreetMap Project (https://osmdata.openstreetmap.de, accessed on 1 January 2022). Final maps and plates were prepared using CorelDRAW^®^ 2019 software.

### 2.2. DNA Extraction, Amplification and Sequencing

**Figure 2:**
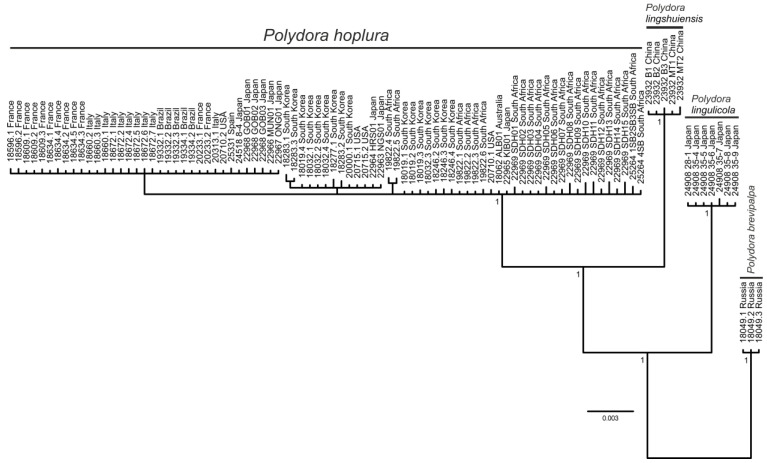
Majority rule consensus tree of the Bayesian inference analysis of the combined *16S* (263 bp), *18S* (1579 bp), *28S* (228 bp) and *Histone 3* (299 bp) sequences (2369 bp in total) of *Polydora* spp. rooted with sequences of *Polydora brevipalpa*. Posterior probabilities of 0.95 and above are shown on the branches. The five-digit numbers preceding collecting locations are unique numbers from the VIR database linking the individuals on the tree with the sampling data in Supplementary Materials Tables S1 and S3; individual numbers are separated from sample numbers by dots.

**Figure 3:**
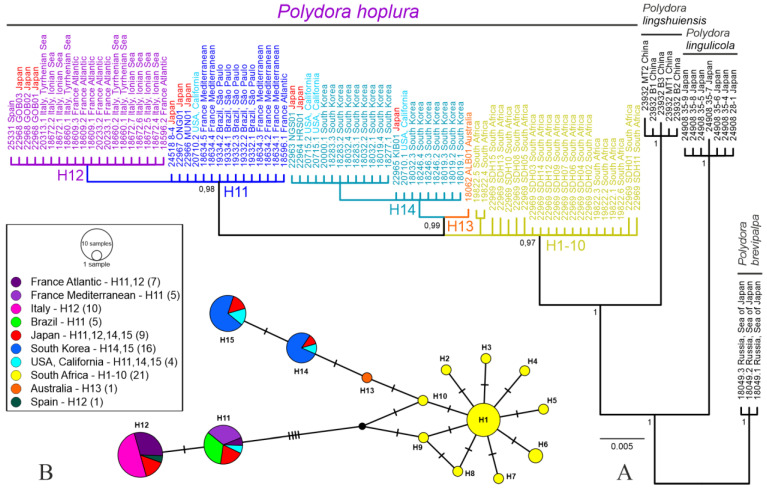
(**A**) Majority rule consensus tree of the Bayesian inference analysis of the *16S* rDNA (263 bp) sequences of *Polydora* spp. rooted with sequences of *Polydora brevipalpa*. Posterior probabilities of 0.95 and above are shown on the branches. The five-digit numbers preceding collecting locations are unique numbers from the VIR database linking the individuals on the tree with the sampling data in Supplementary Materials Tables S1 and S3; individual numbers are separated from sample numbers by dots. Colors indicate the unique South African haplotypes (H1–H10), most common shared haplotypes (H11, H12, H14, H15), and places with most diverse haplotypes (USA, California—3 haplotypes; Japan—4 haplotypes). (**B**) Haplotype network of the *16S* rDNA gene in *Polydora hoplura*. Haplotypes are represented by colored circles whose size is proportional to the number of corresponding individuals. Multiple colors indicate haplotypes shared by individuals from more than one sampling locality, with sections scaled according to their frequency. Each hatch-mark on the lines connecting haplotypes symbolizes one mutational step. A small black circle represents a missing haplotype. Total number of examined individuals shown in parentheses after the names of localities.

We used the ReliaPrep gDNA Tissue Miniprep System (Promega Corporation, Madison, WI, USA) for DNA extraction and purification with standard protocol for animal tissue. Polymerase chain reaction (PCR) amplification of mitochondrial *16S* rDNA and nuclear *28S* rDNA, *18S* rDNA and *Histone 3* gene fragments were accomplished with the primers and the conditions described by Radashevsky et al. [26,27]. In addition, we used the D1R/D2C primer pair to amplify *28S* rDNA gene in some samples [28]. Purified PCR products were sequenced in both directions by the GnC Bio Company, Republic of Korea (www.gncbio.kr), and in the National Scientific Center of Marine Biology, Vladivostok, Russia, using the same primers as for PCR. Sequence editing and contig assembly were performed using SeqScape 2.5 (Applied Biosystems). GenBank accession numbers and brief information about sequences used in the present analysis are shown in Supplementary Materials Tables S3 and S5. To link sequences with complete corresponding data, unique numbers from the first author’s database (VIR) are given to samples in Supplementary Materials Tables S1 and S3. These numbers precede the collecting location names on the phylogenetic trees shown in Figure 2 and Figure 3A.

### 2.3. Data Analysis

In addition to new sequences obtained in the present study, we also included in the analysis sequences of *Polydora lingshuiensis* Ye et al., 2015 from China and *Polydora lingulicola* Abe and Sato-Okoshi, 2020 from Japan provided by Ye et al. [29] and Abe and Sato-Okoshi [23], respectively. These species were sequenced for the most complete set of gene fragments, including *16S*, *18S* and *28S*, and were used to evaluate genetic divergences between *Polydora* species and between distant populations of *P. hoplura*. The phylogenetic tree was rooted using the sequences of *P. brevipalpa*; this species was also sequenced for the most complete set of gene fragments (see Supplementary Materials Table S3).

We aligned DNA sequences using the MAFFT v7.2 software with the default settings (automatically chosen algorithms) [30,31]. Ambiguous positions and gaps for *16S* rDNA, *18S* rDNA and *28S* rDNA genes were excluded from subsequent analysis using trimAl 1.2 [32] with an automated heuristic approach. As the obtained sequences were similar, we chose to employ uncorrected values of sequence divergence (pairwise distances, *p*, see Nei and Kumar [33]) instead of complex distance measures (i.e., corrected by best-fit evolutionary model). Distances both within and between groups were calculated in MEGA 11.0 [34]. We concatenated DNA data partitions using SequenceMatrix 1.9 [35] and specified substitution models for each partition individually. The best-fitting nucleotide substitution models for Bayesian inference (BI) were selected in MrModeltest version 2.3 [36] using Akaike Information Criterion (AIC): SYM + G for each data partition.

We used MrBayes 3.2.7 [37,38] via the CIPRES web portal [39] for the Bayesian analysis of 10,000,000 generations, four parallel chains and sample frequencies set to 1000, in two separate runs. Based on the convergence of likelihood scores, 10% sampled trees were discarded as burn in. The same conditions were implemented for separate BI analysis of *16S* rDNA sequences.

The haplotype network was constructed based on *16S* rDNA dataset with a median-joining approach [40] using popART [41].

## 3. Results

### 3.1. Molecular Analysis

#### 3.1.1. Phylogenetic Analysis of *Polydora Hoplura*

The combined aligned sequences of *Polydora* spp., with gaps excluded, comprised a total of 2364 bp, including 259 bp (98.5% of original sequences) for *16S* rDNA, 1578 bp (99.9% of original sequences) for *18S* rDNA, 228 bp (99.1% of original sequences) for *28S* rDNA and 299 bp (100% of original sequences) for *Histone 3*. The combined concatenated dataset contained 146 variable sites, 141 of which were parsimony informative; the frequencies of variable and informative sites were 6.17% and 5.96%, respectively. The frequency of variable sites in the aligned dataset of mitochondrial *16S* rDNA (19.7%) was greater than those in sequences of the nuclear genes: 2.72% for *18S*, 4.38% for *28S* and 13.37% for *Histone 3*.

The Bayesian analysis of the combined dataset of four gene fragments and the analysis of only *16S* rDNA sequences both showed that individuals from distant localities with diagnostic morphological features of *P. hoplura* form a well-supported clade (PP = 1 and PP = 0.97, respectively) (Figure 2 and Figure 3A). The maximum uncorrected *p*-distance value for the most variable mitochondrial *16S* rDNA between individuals from different populations (*p* = 2.54) was comparable with the maximum genetic variability within these populations (*p* = 1.31) and essentially smaller than the minimum distance between *Polydora* species (*p* = 6.98) (see Supplementary Materials Table S4). Therefore, we consider the examined populations as conspecific and refer them to *P. hoplura*.

#### 3.1.2. Haplotype Network of *Polydora Hoplura*

The haplotype network analysis was based on 79 sequences of *16S* rDNA, of which 26 were previously obtained by Sato-Okoshi et al. [21], Abe and Sato-Okoshi [23] and Almón et al. [24]. The analysis recovered a single network comprising 15 haplotypes (Figure 3B, Supplementary Materials Table S5). Striking 10 unique haplotypes (H1-10; 21 individuals) were found only in South Africa’s Western Cape. They formed a star-like structure with nine haplotypes (H2–H10) differing from the most common haplotype (H1, comprising 52% of South African individuals) by one mutational step. The majority of other individuals shared from one to four of the four most common haplotypes H11 (19%), H12 (25%), H14 (11%) and H15 (16%). Each of these haplotypes was detected in nine individuals from Japan; three of these haplotypes (H11, H14, H15) were detected in four individuals from California. All ten examined individuals from Italy (four from the Tyrrhenian Sea and six from the Ionian Sea) shared one haplotype (H12), which was also detected in the six individuals from Atlantic France (five from Arcachon Bay and one from Brittany, La Manche), three individuals from Japan and in one individual from Galicia, Spain. The haplotype H13 was detected only in one examined individual from Western Australia (LC101868, [21]). Two shared haplotypes (H14, H15) were detected in 16 individuals from Republic of Korea. Only one shared haplotype (H11) was detected in all five individuals from Brazil, all five individuals from Mediterranean France (Gulf of Lion) and in one individual from Atlantic France (Brittany, La Manche).

### 3.2. Morphology and biology

***Polydora hoplura* Claparède, 1868**

Figure 4

**Figure 4:**
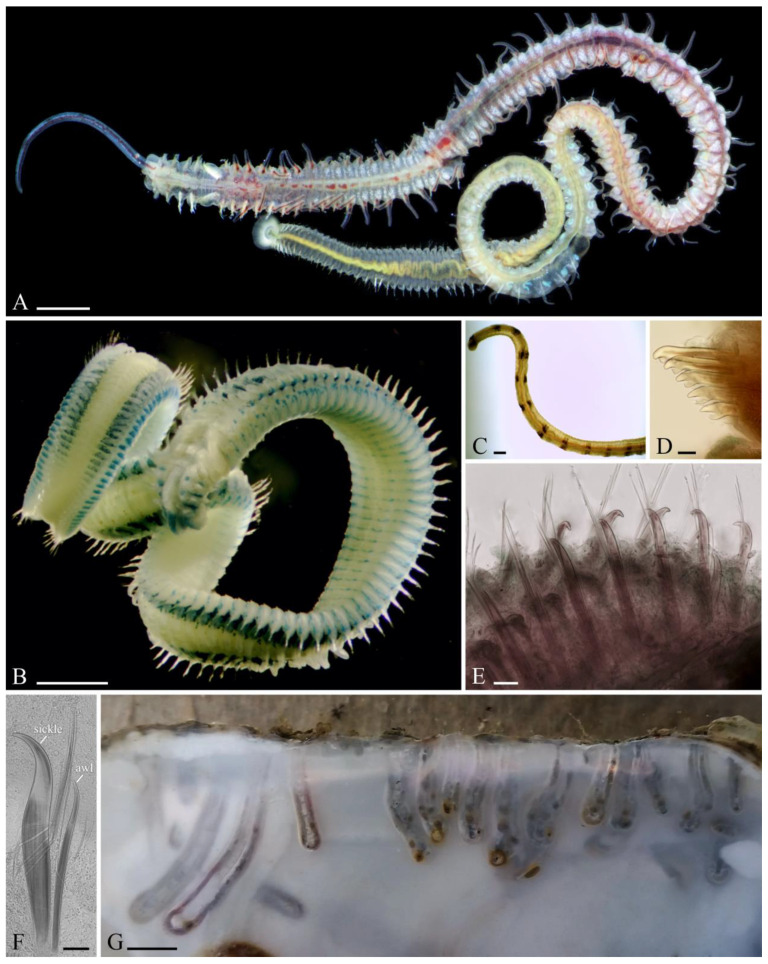
Adult morphology and habitat of *Polydora hoplura*. (**A**) Complete live individual, dorsal view; left palp missing. (**B**) Complete individual fixed in formalin and stained with methylene green; palps missing. (**C**) Palp fragment, showing paired black bands on sides of frontal groove. (**D**) Heavy falcate spines of chaetiger 5, left side, ventral view. (**E**) Posterior notopodia with heavy recurved spines in addition to slender capillary chaetae, left lateral view. (**F**) Two kinds of spines (heavy sickle-shaped and slender awl-like) and slender capillary chaetae from a posterior notopodium. (**G**) Edge of a shell of the Pacific oyster *Magallana gigas* showing U-shaped burrows of worms. Scale bars: (**A**), (**B**)—1 mm, (**C**)—200 µm, (**D**)—30 µm, (**E**), (**F**)—50 µm, (**G**)—5 mm. (**A**)—California, USA (LACM-AHF Poly 12859); (**B**)—Mar Grande of Taranto, Italy (MIMB 33029); (**C**), (**F**)—Wando, Republic of Korea (MIMB 33038, 33035); (**D**)—Gulf of Naples, Italy (MIMB 28148); (**E**), (**G**)—California, USA (LACM-AHF Poly 12862, MIMB 39108). (**A**)—photo by Leslie H. Harris.

*Polydora hoplura* Claparède [42] pp. 318–319, pl. XXII, figure 2; [43] pp. 58–59, pl. XXII, figure 2; [44] pp. 58–59, pl. XXII, figure 2. Andreu [45] pp. 87–91. Leloup and Polk [46] pp. 71–72. Gravina et al. [47] p. 165. David et al. [48] pp. 890–894, figures 4–6 (larval morphology). Radashevsky et al. [12] pp. 545–551, figures 2–4 (references). Radashevsky and Migotto [13] pp. 860–865, figures 2–5 (adult and larval morphology). Sato-Okoshi et al. [21] pp. 1677–1680, figures 6 and 7. Lee et al. [49] pp. 461–463, figure 2. Abe and Sato-Okoshi [23] p. 52, figure 8F. Almón et al. [24] p. 3, figures 1 and 2.

*Polydora* (*Polydora*) *hoplura*: Rioja [50] (*Part*.) p. 70, pl. 19, figures 8–13. Hartmann-Schröder [51] p. 305; [52] p. 318.

*Polydora hoplura hoplura*: Day [53] p. 468, figure 18.2k–m.

*Leucodora sanguinea* Giard [54] pp. 71–73. *Fide* Dollfus [55] p. 17; [56] p. 275.

*Polydora uncinata* Sato-Okoshi [57] pp. 278–280, figure 1; [58] p. 835. Radashevsky and Olivares [15] pp. 491–494, figures 2–4. Sato-Okoshi et al. [59] pp. 493–495, figures 2 and 3; [16] p. 87, figure 4A,B,D. Sato-Okoshi and Abe [17] pp. 43–44, figure 3. *Fide* Radashevsky et al. [12] p. 545.

#### 3.2.1. Diagnostic Features

The adult morphology of *P. hoplura*, including the neotype and other individuals from the type locality in the Gulf of Naples, Italy, was recently redescribed by Radashevsky et al. [12]. Worms from Chile, Republic of Korea and Brazil were described in detail by Radashevsky and Olivares [15] (as *P. uncinata*), Radashevsky et al. [12], and Radashevsky and Migotto [13], respectively. Worms from Japan and South Africa were described by Sato-Okoshi et al. [21]. The diagnostic features of the adults include palps with black bands, prostomium weakly incised anteriorly, caruncle extending to the end of chaetiger 3, short occipital antenna, and two kinds of spines (heavy sickle-shaped and slender awl-like) in addition to slender capillary chaetae in the posterior notopodia, and cup-shaped to disc-like pygidium with middorsal gap (Figure 4A–E). Moreover, all the populations examined by us were parthenogenic (Radashevsky unpublished). Some characters are variable, however. Pigmentation on palps and anterior chaetigers can be well developed or totally lacking. Prostomial incision in some individuals is weakly developed and can be seen in the ventral view only or not developed at all. The caruncle is short in small worms and the occipital antenna is occasionally small and hardly discernible. Slender awl-like spines in the posterior notopodia are arranged differently in individuals of different sizes, and in large individuals are present in a few posterior branchiate chaetigers only [12]. None of these characters is unique; all of them are shared by some other spionids. Nevertheless, the combination of these characters defines the species unambiguously, and the heavy sickle-shaped spines in the postbranchiate chaetigers are the most diagnostic. Such types of spines are also present in *Polydora colonia* Moore, 1907 [60], some *Boccardiella* and some other spionids [61], which, however, can easily be distinguished by other characters.

#### 3.2.2. Habitat

Adults of *P. hoplura* make U-shaped burrows in shells of barnacles, bivalves and gastropods, including clams, oysters and cultured abalone (Figure 4F). Occasionally, worms bore into sponges [13,20,62], coralline algae [20,62,63,64] and the stony coral *Mussismilia hispida* (Verrill) (present study) (Supplementary Materials Table S1). Branches of each U are situated close to each other and interconnected by a narrow space all along their length, thus the burrow in cross-section appears as a characteristic 8-shaped hollow in a shell. The walls of the burrows are lined with detritus; the median space between branches is also filled with detritus forming a medial detrital wall. Each burrow opens to the outside via two joined apertures and is extended by two smooth silty tubes, each up to 5 mm long.

#### 3.2.3. Remarks

The larval morphology of the species was described from the Plymouth area (English Channel, UK) [65], Chile [15] (as *P. uncinata*) and Brazil [13]. Poecilogony with females producing two kinds of larvae, planktotrophic and adelphophagic, was demonstrated in South African populations [48].

Infestation of cultured oysters and especially abalone by *P. hoplura* has been a serious problem for aquaculture for a long time. Commercial interest stimulated studies on the morphology, genetic structure, reproductive biology and dispersal ability of this species [9,19,20,48,49,66,67,68,69,70,71,72,73,74]. Some control of polychaete pests on cultured molluscs has been suggested [67,75,76,77,78,79,80], but the problem of infestation continues.

#### 3.2.4. Distribution

Complete information about worldwide records of *P. hoplura* is provided in Supplementary Materials Table S1 (mapped on Figure 1).

## 4. Discussion

### 4.1. Population Genetics of Polydora hoplura

We used the set of genes originally chosen for phylogenetic analysis of Spionidae [27,81]. The *16S* rDNA is the most variable among examined markers and has been proven to be highly suitable for molecular identification at the species level for spionids [26,27,81,82,83,84,85,86]. Our genetic analysis of *16S* dataset, however, does not reveal strongly robust biogeographic patterns of *P. hoplura* populations worldwide, since haplotype analysis was not the primary objective of our study, nor were our analyzed sample sizes large enough to reveal the full extent of haplotype diversity in any one region.

### 4.2. Historical and Modern Biogeography and Vectors of Dispersal

Potential explanations of the observed haplotype network are confounded in part by the majority of our analyzed specimens representing worms recovered from cultured oysters and abalones (in sea farms, in sea cages, or land tanks) (Supplementary Materials Table S1). These cultured molluscs likely represent, in most cases (even in Japan), populations that have been mixed and remixed for many decades through aquaculture movements and (for oysters) historical transport in vessel-fouling communities (as we discuss below). Our sequenced material from Italy and Brazil was from natural substrates and our material from Atlantic France was from both natural substrates and commercial oysters, while the rest of our sequences are derived from commercial molluscan populations.

That said, we suggest that our preliminary data, when combined with historical knowledge of *P. hoplura*’s absence in certain regions, may hint at its home port—its potential endemic region—even as our data also reveal an intriguing concentration of unique haplotypes in one region with a relatively new, but intensive, history of imported mollusc culture. We briefly review the global history of *P. hoplura* before suggesting a pathway to its native province (see Table 1).

*Polydora hoplura* was first collected in 1866–1867 in Italy [42] in barnacle shells. It was found in many different habitats in the decades thereafter, or had already spread before being collected in Italy, in the northern and western Mediterranean and in Western Europe. It was first found in 1946 in South Africa in Langebaan Lagoon [89] and in 1947 in Cape Town fouling communities [90]. After a long but not unusual hiatus typical of the global spread of marine invasions [91], it was collected in 1971 in Australia (Supplementary Materials Table S1) and in 1972 in New Zealand [92], followed by another multidecade gap before being recovered in Brazil in 1995 (Supplementary Materials Table S1) and again in 2015 [13], in Japan in 1997 [57], in Chile in 2002 [15] and in Republic of Korea and California in 2004 (Supplementary Materials Table S1). In all areas of the world, *P. hoplura* is now established in the wild, except for Chile and perhaps Republic of Korea, as discussed below.

*Polydora hoplura* has long been recorded from molluscan hosts and other calcareous substrates (such as barnacles and serpulid tubeworms) as well as from fouling communities on docks and pilings (Supplementary Materials Table S1). This broad habitat diversity, combined with its known larval biology, suggests three probable human-mediated global transport vectors: (1) the movement of commercial shellfish, (2) vessel fouling and (3) vessel ballast water.

Relative to (1), the Pacific oyster *Magallana gigas* (Thunberg) (commonly known as *Crassostrea gigas*) is one of the most “globalised” species dominating bivalve production in many regions [6,93]. Transoceanic movement of *M. gigas* commenced over 100 years ago [93]. Native to the cool-to-warm temperate shallow waters of the Asian Pacific (Japan, Republic of Korea and Russia), *M. gigas* “have been introduced to 66 countries outside their native range, mainly for aquaculture, and there are now established self-sustaining populations in at least 17 countries” [94] (p. 2836). Biofouled adult oysters were typically transported long distances (to establish new populations or for commercial outplanting) both historically [95] and into modern times [96], providing ample opportunity for numerous accompanying nontarget epibiotic and endobiotic species to be introduced.

Relative to (2), classic hull-fouling communities on both coastal and ocean-going vessels include abundant calcareous substrates such as barnacles, oysters, serpulid polychaetes and also noncalcareous habitats such as sponges [97]. Spionid polychaetes, including *Polydora* spp., have been found in the fouling communities of ocean-going and coastal vessels [98,99,100], (JTC personal observations 2000). *Polydora hoplura* is commonly found in fouling communities on harbor pilings and docks (as reviewed below, and in Supplementary Materials Table S1), which we interpret here to represent individuals likely dislodged from their calcareous substrates or from sponges.

Relative to (3), ballast water may have provided an additional means of dispersal for *P. hoplura* (as we detail further below). *Polydora* larvae have been regularly found in ballast water [101,102,103,104], (JTC and VIR personal observations), with Smith et al. [105] reporting that larval spionids dominated the polychaete fraction in the ballast water of vessels arriving from around the world in Chesapeake Bay. In turn, David et al. [48] found that the average larval life of planktotrophic larvae of *P. hoplura* is 40 days, sufficient time (for those populations with planktotrophic larvae) for both transoceanic and interoceanic transport of larvae in ballast water.

Given the historical records of *P. hoplura*’s reports around the world, combined with the known range of potential anthropogenic vectors, we review here the probable interplay of the chronological history of reports with the probable transport vectors at the times and places of discovery (see Table 1).

*Polydora hoplura* was found in Italy and France prior to the introduction of commercial molluscs from elsewhere in the world [93,106]. We suggest that it arrived in the Mediterranean in vessel fouling, perhaps in barnacles, serpulid tubeworms or oysters attached to hulls. Importantly, ballast water was not a common vector for species in international vessel traffic until the 1880s and later [107]. Its spread around the European theater may then have been facilitated by coastal vessel traffic (both fouling and, later, ballast water) and by the intracountry movement of commercial oysters. Its occasional appearances in the Netherlands [108] and Belgium [109] have been suggested to be directly linked to oyster importations.

The appearance of *P. hoplura* in the late 1940s in South Africa similarly predated the introduction of commercial molluscs, with the importation of the Pacific oyster *M. gigas* not commencing until 1950 [93]. During and after World War II, there was increased commercial and military vessel traffic connecting Cape Town, Western Europe and Asia, suggesting that shipping provided a steady source of *P. hoplura* (albeit sources not yet supported by genetic connectivity, due, as we suggest, below, to population genetic undersampling in Europe and the Western Pacific).

The first collection of *P. hoplura* in 1971 in Australia followed a long history of the importation of the Pacific oyster *M. gigas* from Japan commencing in the 1940s to Victoria, Tasmania, Western Australia, and New South Wales [93]. The first collections of *P. hoplura* at about the same time, in 1972, in southern ports of New Zealand’s North Island, link to a more complicated vector history. Dinamani [110,111] suggested that Japanese vessels with oyster fouling accidentally introduced *M. gigas* to the northern part of the North Island in the early 1960s, followed by its southward spread. Earlier, however, Dromgoole and Foster [112] suggested that *M. gigas* may have been intentionally introduced. The location (Wellington Harbor), habitat (native wild pen shells, oysters, scallops and abalones), and timing (1972–1974) of the first collections of *P. hoplura* in New Zealand are not, however, clearly related to the first appearance of alien oyster populations in the same area, but suggestive of the introduction of *P. hoplura* by vessels.

*Polydora hoplura* was next collected in Brazil in the native stony coral *Mussismilia hispida* in 1995 at Ilha de Alcatrazes and in shells of the native oyster *Crassostrea rhizophorae* (Guilding) (Supplementary Materials Table S1) and in fouling on pier pilings in 2015 [13]. *Magallana gigas* introductions began in Brazil in 1974, resumed in 1986, and importations continue [96]. While the collection sites of *P. hoplura* in Brazil (São Paulo) are not oyster culture sites, *M. gigas* has been imported and outplanted both to the north and south of Sao Sebastião and Alcatrazes [96].

*Polydora hoplura* was transported from Japan to Coquimbo, Chile, perhaps commencing as early as 1987 [15], in the shells of the abalone *Haliotis discus hannai* Ino imported for land-based culture. *Polydora hoplura* were found in these abalones in 2005. Radashevsky and Migotto [13] noted that the “progeny of the abalone were … going to be introduced into coastal waters for further commercial cultivation”, the possibility of which was also discussed by Stotz et al. [113], but there is no evidence that any abalones were ever released into the sea (JTC based on a visit to and interviews in Coquimbo in 2019).

In Republic of Korea, *P. hoplura* was first found in 2004 in cultured oysters *M. gigas* and tank-cultured abalone *Haliotis discus discus* Reeve presumably imported from Japan in 2002; Sato-Okoshi et al. [16] noted that it had been absent prior to 2004. It is also now found in Republic of Korea in abalones cultured in cages in the sea [12] (Supplementary Materials Table S1).

*Polydora hoplura* was first found in California in 2004 (Supplementary Materials Table S1), and collected again in 2011 [13], all in biofouling communities on docks and pilings. It was found again in 2017 in the shells of *M. gigas* in the wild on a pier and in a commercial oyster farm (Supplementary Materials Table S1). Crooks et al. [114] reviewed the history of the discovery of wild populations of *M. gigas* in southern California commencing in 2000, likely linked to unreported and unrecorded aquaculture introductions. Its presence to the north in Monterey Bay in 2011 is likely due to coastal vessel traffic. Among the various global regions in which *P. hoplura* is now established, southern California offers perhaps one of the most robust historical baselines, in terms of polychaete collections. Southern California coastal polychaetes were extensively sampled between the 1930s and 1980s by the well-known polychaetologists Olga Hartman [115,116], Keith Woodwick [117] and Donald Reish [118,119], and it is highly unlikely that these careful workers would have overlooked *P. hoplura* in biofouling communities, which they regularly sampled.

In summary (Table 1), we suggest a chronological progression of the driving global dispersal vectors of *P. hoplura*. The earliest record of *P. hoplura*, in what appears to be its first long-distance colonization episode, we relate to vessel fouling, before the advent of the international movement of ballast water and of commercial oyster movements. Following widespread dispersal through Western Europe, it was not until the mid-20th century that *P. hoplura* was found in South Africa, by which time both vessel fouling and ballast water were in play. While we do not set aside the continued role of vessel traffic, we relate the appearance of *P. hoplura* in Australia, Brazil and California to the vast uptick of the movement of Pacific oysters since the 1970s around the world, with vessel fouling and ballast water likely playing roles in the secondary movement of populations (Table 1). The evidence in hand does not permit us to separate out the relative roles of vessel traffic *versus* oyster culture in the arrival of *P. hoplura* in New Zealand.

### 4.3. Maritime History Considerations and Invasion Timing

In the 1730s and 1740s, the East Indiaman cargo vessel *Schellak* made regular voyages from the Netherlands around South Africa to Jakarta in Indonesia, with occasional trips as far north as Japan [120]. By the late 1700s, regular passages by ships had commenced between Britain and Australia [121]. Could hundreds—and eventually thousands—of such early maritime voyages, fluidly connecting Europe, South Africa, the Indian Ocean, Australasia, and the North Pacific, have transported and introduced marine worms many centuries ago? Could *P. hoplura* specifically have been introduced by ships to South Africa and Australia as early as, for example, the 1700s, and overlooked in these countries until the 20th century?

There is no doubt that ocean-going vessels moved and introduced marine animals and plants interoceanically, often undetected, for hundreds of years, resulting in the obfuscation of the historical biogeography of a great many species [10,122,123,124,125]. That said, a great many variables contribute to an often strong disconnect between the existence of a vector and the probability of successful introductions [126], as witnessed by continued new invasions of many biofouling species around the world, despite the fact that the same species have been carried around the world over many centuries.

*Polydora hoplura* is a comparatively large, distinctive worm, not easily overlooked; it reaches 55 mm in length and bears large conspicuous recurved spines in the posterior notopodia, easily recognized by polychaete workers [12]. *Polydora hoplura* also has broad habitat diversity, being found in a wide range of calcareous substrates (molluscs, tubeworms, and barnacles) and in sponges. Collecting *P. hoplura* is not limited to examining calcareous substrates; biofouling collections from harbor docks and pilings produce individuals dislodged from their substrates (Radashevsky unpublished).

In both South Africa [127] and Australia [128], collections of shallow-water marine worms began in the 1850s and were well underway by the turn of the 20th century. In Australia, the zoologist William Haswell worked out of a laboratory on the shores of Sydney Harbour commencing in the 1890s [128]. Haswell [129], Whitelegge [88] and Roughley [130] investigated oyster-boring spionids, reported as *Polydora ciliata*, in Australia. Blake and Kudenov [131] suggested that Haswell’s and Whitelegge’s reports may refer to *Polydora websteri* Hartman in Loosanoff and Engle, 1943. As we note in Table 1, Carazzi [87] suggested that Whitelegge [88] had misidentified *P. hoplura* as *P. ciliata*, but Whitelegge’s description and figures suggests that he had neither species. Roughley [130] reported boring worms in oysters, also identified as *P. ciliata*, but no longer than 2.5 cm, indicating that these were not *P. hoplura.* Extensive polychaete work in Australia resumed in the 1950s and 1960s, without *P. hoplura* being reported. Thus, for Australia, we have reasonably thorough indications that *P. hoplura* was not likely present prior to the mid-20th century. In South Africa, worm collections in the Cape Town area were underway by the early 1900s, without the conspicuous *P. hoplura* being reported. Day [132] specifically reported on spionids in South Africa, and, again, this conspicuous worm was not recorded.

In clear contrast, as we have detailed above, large numbers of Pacific oysters, a well-known host of *P. hoplura*, began to be imported in the 20th century into both Australia and South Africa. We suggest it is not a coincidence that these commercial aquaculture operations were a major vector in introducing *P. hoplura* to these countries.

We recognize a distinction between when a non-native species is first collected and when it may have been introduced. In many cases there are well-known lag times between initial arrival and colonization of a species and its population growth to the point where it can be detected (collected) [133]. Nevertheless, for species that are relatively large and conspicuous in regions with a relatively long history of investigation, the lag time between a species becoming established and encountering it in the field is rarely on the order of centuries, except for those invasions going unrecognized under other names. We have no evidence that this is the case for *P. hoplura* in the regions where it has now been found.

This said, we do not know when *P. hoplura* first arrived in the Mediterranean: it may have been introduced to the Gulf of Naples well before the 1860s. Similarly, South African populations may of course have been present some years before 1946, and Australian and New Zealand populations may have become established by the 1960s. None of these adjustments would alter the larger picture that *P. hoplura* is a species whose global voyages commenced and continued through the 19th and 20th centuries.

Finally, the uptick in the globalization of *P. hoplura* commencing in the last half of the 20th century and continuing into the early 21st century falls into a now well-recognized pattern of a world sustaining more and more invasions paralleling a significant parallel uptick in maritime trade. Examples abound: the well-known European shore crab *Carcinus maenas* (Linnaeus), although introduced by ships to Atlantic North America and Australia in the 19th century, began resuming its global voyages in the late 20th century [91]. The Asian mitten crab *Eriocheir sinensis* H. Milne Edwards, introduced to Europe in the early 1900s, also did not resume its voyages until the late 20th century [134]. The abundant intertidal barnacle of the Northeast Pacific, *Balanus glandula* Darwin, despite hundreds of years of opportunity to spread around the world, only began colonizing foreign shores (Argentina, Japan, South Africa) in the late 20th century [135]. The Japanese caprellid amphipod *Caprella mutica* Schurin similarly began spreading globally only in the 1970s [136]. There are scores of additional cases. We suggest that the increasing number of recognized populations of *P. hoplura* nests well within this modern pattern of increased global invasions.

### 4.4. Possible Native Range of Polydora Hoplura

We suggest that the global history of detection of *P. hoplura*, combined with the albeit limited haplotype data in hand, permit preliminary identification of its potential native region. Haplotypes H11, 12, 14 and 15 are found in Japan. We know that *P. hoplura* appeared in approximately 2004 in Republic of Korea, and the presence of H14 and 15 there, combined with the history of importations of *M. gigas* from Japan, suggest that Republic of Korean populations are derived from there. California populations possess H11, 14 and 15, as does Japan. Italy and Atlantic France possess H12, as does Japan. Brazil populations to date present the H11 haplotype, of yet uncertain origin. In short, the shared haplotypes in the North Pacific, versus only two shared haplotypes (H11, H12) detected in the Northeast Atlantic and Mediterranean (both of which also occur in the North Pacific), suggest that *P. hoplura* originated in the Pacific Ocean, not in the Mediterranean Sea. The demonstrable historical absence of *P. hoplura* from California (above), versus our inability to demonstrate the same for Japan, narrows the possible origin to the Western Pacific, or at the most the Indo–West Pacific.

The late detection of *P. hoplura* in Japan may be explained by the lack of earlier extensive studies within the potential range of this species. The shell-boring spionids in Japan were first studied in the scallop *Mizuhopecten yessoensis* (Jay) cultured in northern Hokkaido and northeastern Honshu [137,138,139,140], areas likely too cold for the warmer water *P. hoplura*. Prior to the 1990s, there is only one report of shell-boring spionids in southern Japan (Tokushima Prefecture, Shikoku Island), that of Kojima and Imajima [141], who identified *Polydora ciliata* and *Polydora websteri* in the wild abalone *Haliotis diversicolor* Reeve. Sato-Okoshi [57] described *P. uncinata* (=*P. hoplura*) burrowing in shells of the wild gastropod *Omphalius rusticus* (Gmelin) from Kochi Prefecture (Shikoku Is.) and in cultured *M. gigas* from Iwate Prefecture (Honshu Island). Soon after that, Sato-Okoshi [58] reported 13 shell-boring polydorins from Japan, including seven *Polydora* species.

We are left with the apparent enigma of the intriguing genetic diversity of *P. hoplura* in South Africa. All 10 *16S* haplotypes detected in the Western Cape Province were unique and not yet recovered in other global populations. David et al. [19] recovered 42 haplotypes (seven shared, 35 unique) for the mitochondrial *cytochrome b* (*Cyt b*) gene, and 44 haplotypes (14 shared, 30 unique) for the ATP-synthetase nuclear-encoded protein complex (ATPSα) gene from seven localities encompassing the reported distributional range of *P. hoplura* in South Africa. No geographic pattern of haplotypes was detected in that study; frequent movement of oysters along with coastal shipping (such as worms burrowing into sponges and barnacles on ship hulls) were suggested as factors homogenizing South African populations [19] (p. 608) [142,143]. In addition, we note that documenting unique haplotypes in introduced populations—that is, genes not yet detected in the known home range of a species—is a recognized phenomenon [144,145,146].

South African populations of *P. hoplura* could thus reflect 75 or more years of introductions from around the world via vessel hull fouling and oyster importations. In particular, many decades of oyster imports from a host of different countries would serve to both “collect” and concentrate many haplotypes not yet detected elsewhere. Williams et al. [20] (text and especially their Figure 5) have underscored the scale of anthropogenic introductions of *P. hoplura* in South Africa from potentially many genetically distinct source populations. Our current sample sizes from Europe, Australia, Japan, and elsewhere are too small to capture the global *16S* haplotype diversity of *P. hoplura*. We thus make two predictions: one, that more thorough modern and historical (museum) sampling (below) will reveal the presence in the Pacific Ocean of the 10 haplotypes currently registered only for South Africa (as well as H13 in regions outside of Australia) and, two, that sampling wild native host populations will reveal allochthonous haplotypes in South Africa already known from elsewhere. Our predictions aside, the concentration of haplotypes in South Africa and in the Pacific would suggest at the very most an Indo–West Pacific, if not specifically a Northwest Pacific, origin for *P. hoplura*, not the Atlantic Ocean or the Eastern Pacific.

## 5. Conclusions

In moving around the world, *P. hoplura* has acquired a rich diversity of hosts, including a wide number of bivalve and gastropod mollusc species, serpulid tubeworms, bryozoans, barnacles, sponges and others. The world’s museums possess equally rich historical resources of all of these substrates from all of the countries discussed here. Along with harvesting and sequencing larger numbers of individuals globally, we predict that thorough mining of these museum resources will produce historical material of *P. hoplura* and further elucidate the global biogeographic history of this globe-trotting worm.

Carlton and Ruiz [147] described many of the complexities involved in analyzing the human-mediated dispersal history of marine species. They noted that species that have likely endured multiple mechanisms of global transport are *polyvectic.* The history of the discovery of *P. hoplura* around the world appears to be intimately linked to global shipping that commenced in the mid-19th century, followed by the advent of the global movement of commercial shellfish in the 20th century, interlaced with continued dispersal by vessels and aquaculture. Many populations over the decades may thus have originated from sustained infusions from widespread populations by both vectors. Given that *M. gigas* has been successfully established in no fewer than 17 countries, of which *P. hoplura* has been detected in only a few of those to date, we predict that it may already be established in many more regions. Further, as global connectivity continues to increase in world trade of both products and seafood, it is likely that novel populations of *P. hoplura* will continue to emerge.

## Figures and Tables

**Table 1:** Possible introduction mechanisms and subsequent anthropogenic dispersal vectors by region, arranged chronologically by year of discovery.

Region	Year First Collected	Possible Method of Initial Introduction	Probable Secondary Anthropogenic Dispersal Mechanisms throughout Continent, Country, or State	Established in Wild?	Comments
Mediterranean	Italy: 1866–1867; France: 1873–1874	Vessel fouling	Fouling, aquaculture, ballast water	Yes	Arrival in Mediterranean preceded importations of Pacific oysters (*Magallana gigas*) and the common use of ballast water internationally
Atlantic Europe: France	circa 1880	Vessel fouling	Fouling, aquaculture, ballast water	Yes	Arrival in Atlantic Europe preceded importations of Pacific oysters and common use of ballast water
South Africa: Langebaan lagoon	1946	Vessel fouling or ballast water	Aquaculture, fouling, ballast water	Yes	Probable arrival in South Africa preceded commercial mollusc importations
Australia: Victoria and South Australia	1971 *	Aquaculture	Vessel fouling; ballast water	Yes	Pacific oyster importations commenced in 1940s
New Zealand: Wellington Harbour	1972	Vessel fouling or ballast water?	Aquaculture; ballast water	Yes	See discussion relative to timing of Pacific oyster introductions
Brazil: São Paulo	1995	Aquaculture	Vessel fouling	Yes	Pacific oyster importations commenced in 1970s
Chile: Coquimbo	2002	Aquaculture		No	Japanese abalone (*Haliotis discus hannai*) importations commenced in 1987
Republic of Korea: Wando	2004	Aquaculture	Aquaculture	Uncertain	Pacific oyster and Japanese abalone importations
USA: California: San Diego Bay	2004	Aquaculture	Vessel fouling (north from southern CA to Monterey Bay)	Yes	Pacific oyster importations prior to 2000

* Carazzi [87] suggested that Whitelegge [88] has misidentified *P. hoplura* as *P. ciliata* in Australia. Our study of Whitelegge’s description and figures suggests that he had neither species.

## Data Availability

All the research data are presented in the text of the paper and in the Supplementary Materials.

## Supplementary Materials

The following supporting information can be downloaded at: https://www.mdpi.com/article/10.3390/biology12060780/s1 which includes the following Tables S1–S6: Table S1. Sampling location data and museum registration numbers of *Polydora hoplura*. Table S2. List of the museums and collections (and their acronyms) holding the examined or reported samples of *Polydora* spp. Table S3. Taxa, sampling location data, museum registration numbers and GenBank accession numbers of sequences obtained in the present study and also provided by earlier authors. Complete information about samples is given in Table S1. Table S4. Uncorrected pairwise average distances (*p*, in %) between populations for *16S* (259 bp), *18S* (1578 bp), *28S* (228 bp), and *Histone 3* (299 bp) sequences. Table S5. *16S* haplotypes recognized in the present study. Table S6. References cited in Tables ESM1, ESM3 and ESM5 [148,149,150,151,152,153,154,155,156,157,158,159,160,161,162,163,164,165,166,167,168,169,170,171,172,173,174,175,176,177,178,179].
